# Effectiveness of a guided multicomponent internet and mobile gratitude training program - A pragmatic randomized controlled trial

**DOI:** 10.1016/j.invent.2024.100787

**Published:** 2024-11-12

**Authors:** Dirk Lehr, Henning Freund, Bernhard Sieland, Lina Kalon, Matthias Berking, Heleen Riper, David Daniel Ebert

**Affiliations:** a Department of Health Psychology and Applied Biological Psychology, Leuphana University, Lueneburg, Germany; b Department of Clinical Psychology and Psychotherapy, Vinzenz Pallotti University, Vallendar, Germany; c Institute of Psychology, Leuphana University, Lueneburg, Germany; d Department of Clinical Psychology and Psychotherapy, Friedrich-Alexander-University of Erlangen-Nuremberg, Erlangen, Germany; e Department of Clinical, Neuro and Developmental Psychology, Amsterdam Public Health Research Institute, VU University, Amsterdam, Netherlands; f Department of Sport and Health Sciences, Technical University of Munich, Munich, Germany

**Keywords:** Gratitude, Positive psychology, Repetitive negative thinking, Internet and mobile intervention, Transdiagnostic

## Abstract

**Objective:**

To investigate the effectiveness of a guided, internet- and smartphone-based gratitude intervention on the transdiagnostic risk-factor ‘repetitive negative thinking’. The multicomponent intervention integrates a variety of gratitude exercises, targeting the cognitive, emotional and behavioural facets of gratitude.

**Method:**

Two hundred adults with pronounced repetitive negative thinking were recruited from the general population. Participants were randomly assigned to either a four-session guided gratitude intervention (*n = 100*) or waiting list (*n = 100*). The primary outcome was repetitive negative thinking three months after randomization, with exploratory assessments at six weeks and six months, the latter just for participants in the intervention group.

**Results:**

Following the intention-to-treat principle, by analyses of covariance (ANCOVA), the gratitude intervention group exhibited significantly lower levels of repetitive negative thinking than controls at three months, with *d* = 0.66, *95* *% CI* [0.37, 0.94] maintained at six-month follow-up. Significant and meaningful beneficial effects were observed in symptoms of depression (*d* = 0.42) and generalized anxiety (*d* = 0.38). These effects were notably stronger in intervention completers who finished at least three sessions.

**Conclusions:**

Results suggest that a multicomponent gratitude intervention is effective at reducing repetitive negative thinking. Multicomponent interventions may be a next step needed to fully realize the potential of gratitude interventions. Such interventions could expand the repertoire of transdiagnostic interventions, especially for repetitive negative thinking. Furthermore, due to its positive connotations, gratitude is a candidate for an indirect intervention aimed at reducing the burden of depression in the general population.

**Trial registration:**

The study is registered in the German Clinical Trial Register (approved primary register of the WHO) as DRKS00006825. The trial protocol can be assessed at: https://www.drks.de/

## Highlights

• Repetitive negative thinking is a risk-factor for common mental disorders.
• A multicomponent gratitude intervention reduces repetitive negative thinking.
• Beneficial effects on symptoms of depression and generalized anxiety were found.
• The gratitude training expands the options for positive mental health interventions.
• Multicomponent design may be the next step for improving gratitude interventions.

## 1 Introduction

Repetitive negative thinking (RNT) has been identified as a risk factor for the development and maintenance of various mental health conditions (McEvoy et al., 2013; Watkins, 2008), including stress (Santee et al., 2023), depression and anxiety (Bell et al., 2023; Wahl et al., 2019) and has been found to explain their comorbidity (Spinhoven et al., 2019). It was introduced as a generic term to capture the common characteristics of worry and rumination (Harvey et al., 2004; Ehring and Watkins, 2008). Ehring et al. (2011, p. 226) summarized these common components – worry and rumination – as “a style of thinking about one's problems (current, past or future) or negative experiences (past or anticipated) that has three key characteristics: the thinking is repetitive; it is at least partly intrusive; and it is difficult to disengage from”.

With the rise of the transdiagnostic perspective, repetitive negative thinking has appeared as a target of interventions (Dalgleish et al., 2020). Newby et al. (2015) have published meta-analytic evidence supporting the efficacy of transdiagnostic treatment at reducing depression and anxiety. There also is meta-analytic evidence suggesting the efficacy of treatments targeting depression at reducing repetitive negative thinking, and that these effects are linked to the interventions' effects on depression severity (Spinhoven et al., 2019). Moreover, initial evidence suggests that reducing repetitive negative thinking may be a mechanism of change in interventions for depression (Domhardt et al., 2021). Recently, transdiagnostic interventions have been investigated in preventative settings, showing promising results for repetitive negative thinking and symptoms of depression and anxiety (Topper et al., 2017; Heckendorf et al., 2022), especially within indicated prevention programs for those experiencing high levels of repetitive negative thinking (Heckendorf et al., 2022; Hirsch et al., 2020; Cook et al., 2019).

There are several reasons to assume that interventions to promote gratitude could be a worthwhile transdiagnostic approach targeting repetitive negative thinking. First, considering effective interventions for reducing repetitive negative thinking, Querstret and Cropley (2013, p. 1006) stated that “interventions which require active mental engagement are useful because active mental engagement interferes with the more passive cognitive processes of perseverative thinking”. Such active mental engagement could involve learning not just to avoid overlooking positive elements, but to remain flexible and actively adopt an overall positive perspective (Lyubomirsky and Tkach, 2003), which may describe features of gratitude interventions.

Second, following this line of reasoning, Watkins (2014) proposed that gratitude interventions – like gratitude journaling and drafting letters of gratitude – could be a promising approach to counteract repetitive negative thinking. There are several potential reasons for this. First, the amplification theory of gratitude (Watkins, 2014) proposes that gratitude amplifies good from one's past, present, and future by different mechanisms. Such mechanisms include enhancing one's ability to reframe unpleasant events positively; increasing accessibility to positive memories; enhancing one's enjoyment of activities, awareness of pleasant events and social relationships; and, most importantly, counteracting rumination.

Third Davis et al. (2016) regard gratitude exercises as highly promising, as participants seem to enjoy them, and find them comparably easy to understand and perform, as well as naturally socially- and other-orientated, thereby focusing on meaningful experiences and memories. Results published by Folk and Dunn (2023) are consistent with this, as they found gratitude to be among the most commonly recommended mental health promotion strategies in the media.

Finally, there is moderate evidence from meta-analyses supporting the efficacy of gratitude interventions against depressive symptoms (Cregg and Cheavens, 2021; Diniz et al., 2023); with randomized controlled trials (RCT) showing promising effects on outcomes related to repetitive negative thinking in terms of ruminative response style in patients with cervical cancer (Shao et al., 2016), anxiety (Kerr et al., 2015), death worry among breast cancer survivors (Otto et al., 2016), and worry in general (Geraghty et al., 2010).

Despite these promising studies, several authors of existing meta-analyses examining the effects of gratitude interventions on mental health have expressed reservations about the enthusiasm for gratitude interventions found in the literature (Davis et al., 2016; Cregg & Cheavens, 2021). Davis et al. (2016) called for interventions that are more effective and concluded that the potential of gratitude interventions might not yet be realized. Starting with a landmark study conducted by Emmons and McCullough (2003), most studies published to date have employed a single gratitude exercise, like a daily or weekly gratitude journal or gratitude letter or visit, and might, thereby, have captured just one aspect of a more complex phenomenon. Considering the conceptual work conducted by Gulliford et al. (2013) and theory building performed by Watkins (2014) and Algoe (2012), it seems reasonable to assume that gratitude is a complex phenomenon that includes at least attentional processes, cognitive processes like appraisal and memory recall, emotional experiences, and action tendencies, especially the actions of (pro)social exchanges. Therefore, to realize the potential of gratitude interventions, it might be promising to develop a next generation of multicomponent interventions that consider the many facets of gratitude. For example, the cognitive, emotional and behavioural facets of gratitude can be addressed by different exercises focusing on the respective facet. Likewise, the positive valence of gratitude is often inextricably linked to indebtedness (Gulliford et al., 2013) and multicomponent interventions should also account for that facet of gratitude. First trials exist that have investigated this approach to intervention design. For example, Bohlmeijer et al. (2021) developed a six-week, mainly-written training program comprised of a variety of exercises. An interactive multicomponent mobile gratitude intervention was investigated by Kloos et al. (2022), who found positive effects for repetitive thinking, and for symptoms of depression and anxiety in the general population. Similarly, Heckendorf et al. (2019) reported comparable effects for GET.ON gratitude training, a combined internet and mobile gratitude intervention consisting of five weekly sessions accompanied by a picture-based mobile gratitude diary.

Considering the potential realization of gratitude interventions, it should be noted that these multicomponent programs, along with most gratitude interventions (e.g., Cregg & Cheavens, 2021), to date have been delivered without personal support. Therefore, it remains unclear to what extent participants could benefit if such interventions were delivered with guidance from a mental health expert. In other research, the same intervention delivered with personal support has been found to yield greater mental health benefits for users (Zarski et al., 2016). To summarize, to date, there remains too few studies investigating the effect of gratitude on repetitive negative thinking using multicomponent and guided interventions. As most gratitude interventions were designed for self-help, the provision of intensive personal guidance can serve as a best-case scenario to explore the magnitude of achievable effects of gratitude interventions.

The present study aimed to test whether the multicomponent program GET.ON Gratitude, provided with personal support, is superior to being on a waiting list with unrestricted access to care as usual at reducing repetitive negative thinking in a sample recruited from the general population. Additionally, effects on secondary mental health outcomes, including symptoms of depression and generalized anxiety, were investigated exploratively.

## 2 Materials and methods

### 2.1 Study design

**Fig. 1:**
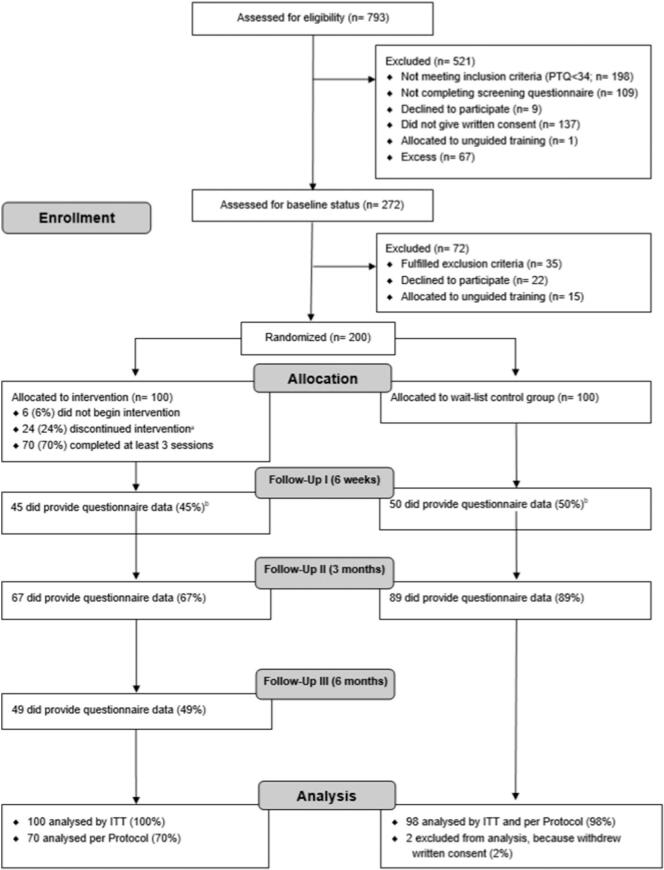
Flow of participants. Follow-up I: Six weeks after randomization; Follow-up II: Three months after randomization; Follow-up III: Six months after randomization. PTQ = Perseverative Thinking Questionnaire; IG = Intervention group; WLC = Waiting-list control group; ITT = Intention-to-treat analysis. ^a^ Participants who completed fewer than 3 out of 4 units. ^b^ Counts/Percentage of provided data for the primary outcome.

The present study was designed as a two-arm, pragmatic, randomized controlled trial (RCT). Two hundred participants were randomly allocated (at a ratio of 1:1) to either a guided smartphone and internet-based gratitude training intervention (GET.ON gratitude) or a waiting-list control group. Both the intervention and control group had full access to care as usual. Outcomes were measured pre-intervention (T1); as well as at six weeks (T2) and three months (T3) after randomization. A final, additional assessment of just those in the intervention group was conducted six months post randomization (T4) (Fig. 1, Flow-Chart).

Repetitive negative thinking at three months was defined as the primary outcome. Based on effects reported in previous intervention studies on gratitude (Davis et al., 2016), prior experience with similar intervention studies, and the intervention only being newly developed, we assumed that the intervention would exert a small-to-medium effect (Cohens *d* = 0.40) on the primary outcome at the three-month assessment, relative to the waiting-list control condition. To test this hypothesis, a total sample size of *N* = 200 was required to achieve 80 % power (1–*β* = 0.80) and 95 % confidence (*α* = 0.05) on two-tailed analysis.

### 2.2 Participants

Participants were recruited nationwide from the German-speaking general population. After the public was informed about the study on gratitude, several women's magazines were interested in the topic, wrote articles on gratitude, and informed their readers about the study, thereby supporting recruitment*.*

Interested individuals registered at the study's website. Subsequently, they were provided with detailed information about the research project and asked to complete an online screening questionnaire that assessed inclusion and exclusion criteria. Inclusion criteria were a) age ≥ 18 years, b) internet access, c) owning a smartphone, and d) elevated repetitive negative thinking, defined as a score > 33 on the Perseverative Thinking Questionnaire (PTQ; Ehring et al., 2011). Individuals who a) were participating in another online-training trial targeting mental health, b) were waiting to receive or currently receiving professional psychological help, c) had changed the dose of some psychopharmacological treatment in the last month, d) reported acute suicidal tendencies (score of <2 on suicidality item from Beck-Depression-Inventory II; Hautzinger et al., 2009), or e) reported dissociative symptoms, were excluded from participation. These criteria were assessed by an online screening questionnaire.

Eligible individuals provided informed consent, completed the baseline assessment, and then were randomly assigned to one of the two study groups. Participants in the intervention group had access to the intervention immediately after randomization, whereas controls were offered access to the intervention shortly after the follow-up assessment three months after randomization. Control group access was not restricted to those who completed all the follow-up assessments. The intervention was free of charge and participants received no financial compensation. Randomization took place from August 2014 to March 2015. To ensure allocation concealment, randomization was conducted by an independent researcher using an automated computer program (randomization.eu). Blinding was not feasible. The study was approved by the Psychological Ethics Committee at Phillips University of Marburg (Nr. AZ-2014-09 k) and registered before the start of participant enrolment as DRKS00006825 in the German Clinical Trial Register, a primary registry of the World Health Organization (WHO). Fig. 1 summarizes the flow of participants through the study.

### 2.3 Intervention

The GET.ON gratitude training program that was offered to participants consisted of four web-based modules and a gratitude smartphone application for daily exercises. Participants were advised to work through one module per week, each of which generally required 45 to 90 min. Each session entailed psycho-educative components, exercises, and testimonials from persona who were created both to encourage participants and as examples of how to cope with obstacles and setbacks. The intervention's content was offered in an interactive and adaptive manner, including videos, audios, and gamification elements.

The intervention was designed along a newly-developed working model of gratitude and mental health called the “upward spiral of gratitude and well-being.” Briefly, this model assumes that potential positive events - gratitude eliciting situations - lead to increased mental and social well-being through different sequential and parallel processes, starting with perception, gratitude-promoting thoughts, the feeling of gratitude, and action tendencies (see supplementary material for graphical representation, S1). The model was used as a framework to integrate 17 different gratitude exercises into a comprehensive, multicomponent gratitude intervention. For example, reminders in the app to take a note or photo of a moment of gratitude aimed to increase the perception of present positive experiences, while working with a life graph aimed to increase awareness of past experiences of gratitude. Exercises for cognitive restructuring and behavioural experiments addressing gratitude-promoting thoughts, a guided imagery exercise aimed to strengthen the feeling of gratitude, and gratitude letters or visits were among the exercises introduced to follow the action tendencies of gratitude. Exercises like gratitude journaling, writing a letter expressing gratitude, and gratitude visits were borrowed from previous literature. Additionally, certain established therapeutic exercises (e.g., working with a life graph) were modified to enhance gratitude. A more detailed description of the exercises included in the program are available in the supplementary material (S2) or as a written manual in Freund and Lehr (2020). The same intervention was used as a pure self-help program without guidance in a study by Heckendorf et al. (2019), but as a five-session version.

### 2.4 Support

Intervention group participants were guided by trained eCoaches, who provided personal feedback after participants completed each training module. For this purpose, the eCoaches reviewed the participants' entries about their experiences with the gratitude exercises, encouraged them to try out new solutions when problems arose, helped them to recognise their own progress, and encouraged them to continue practising the exercises. In addition, eCoaches sent reminders if participants did not complete a module within one week, and were available for any questions that arose. Communication between the eCoaches and study participants was facilitated by an email-messaging system on the secure, training homepage. To ensure a standardized procedure of coaching, all the eCoaches followed a written coaching manual. Moreover, they were advised to not exceed three hours of coaching per participant over the course of the intervention. All eCoaches had a Master's degree in Psychology and were supervised by a Clinical Psychologist.

### 2.5 Measurements

#### 2.5.1 Primary outcome measure

*The Perseverative Thinking Questionnaire (PTQ)* consists of 15 items that assess a person's level of repetitive negative thinking (Ehring et al., 2011). All items are rated on a 5-point Likert response scale ranging from 0 to 4, with the total score ranging from 0 to 60 (higher scores indicating higher levels of perseverative cognition). The items capture the core characteristics of perseverative cognition: repetitiveness, intrusiveness, and difficulty disengaging. Example items are “The same thoughts keep going through my mind again and again” and “My thoughts take up all my attention”. The PTQ has been tested in clinical and non-clinical trials and shown to have high internal consistency (Cronbach's α = 0.95; α = 89 in the present study at baseline). A detailed description of internal consistency and test-retest reliability is provided in supplementary material S3 for all measurements.

#### 2.5.2 Secondary outcome measures

##### 2.5.2.1 Mental health

The German Version of the *Centre for Epidemiological Studies Depression Scale (CES-D)* was used to assess depressive symptoms (Hautzinger et al., 2012). For measuring participants' level of anxiety, the *Generalized Anxiety Disorder Questionnaire (GAD-7)* was applied (Löwe et al., 2008). The *Insomnia Severity Index (ISI)* measured symptoms of insomnia (Bastien et al., 2001). Level of resilience was measured with the *Connor-Davidson Resilience Scale (CD-RISC,* Connor and Davidson, 2003).

##### 2.5.2.2 Personality

*The Gratitude Questionnaire (GQ-6)* was used to assess participants' dispositional gratitude (McCullough et al., 2002). Participants` dispositional optimism was measured with *the Life Orientation Test (LOT-R,* Scheier et al., 1994).

##### 2.5.2.3 Social support

*The Perceived Available Support* subscale of *the Berlin Social Support Scales (BSSS*) was employed to measure emotional and instrumental aspects of perceived social support from others (Schulz and Schwarzer, 2003).

##### 2.5.2.4 Satisfaction

*The Client Satisfaction Questionnaire (CSQ-8)* (Attkisson and Zwick, 1982), specifically a version adapted for internet interventions (CSQ-I), was used to assess participants' perceptions of the intervention's value (Boß et al., 2016).

### 2.6 Statistical analysis

Multiple imputations were employed to handle missing data using the statistical software program R studio (mice package; version 2022.07.2 Build 576). Based on all available data for variables displayed in Table 2, 20 estimations were calculated per missing value. Pooled and adjusted mean values and standard deviations are reported according to Rubin's Rule (Rubin and Hunter, 1987).

All analyses were conducted adhering to the Consolidated Standards of Reporting Trials (CONSORT) statement following the intention-to-treat principle (ITT). Main and additional study-completer sensitivity analyses, involving only participants who provided data at follow-up, were performed using R studio (version 2022.07.2 Build 576). A two-tailed significance level of 0.05 was used for all analyses.

To test the assumed superiority of the intervention over the waiting-list control condition at three-month follow-up for the primary outcome, we conducted analysis of covariance (ANCOVA), with baseline levels of repetitive negative thinking as the covariate, as recommended by O'Connell et al. (2017). Cohen's *d* and its corresponding 95 % confidence interval (95 %-*CI*) were computed for differences in marginal means between the intervention and control group at each time point using the respective pooled standard deviation. For within-group effect sizes, retest-reliability was considered (Borenstein et al., 2009). The same analytic procedure was employed for all secondary outcomes at T2 (six weeks) and T3 (three months). Additionally, to test the stability of the effects at T4 (six months), we conducted repeated-measure analysis of variance (ANOVA) and calculated within-group effect sizes for all outcomes.

Intervention response rates were analysed from different perspectives. First, reliable improvement and deterioration were determined following the recommendations of Jacobson and Truax (1991), resulting in a Delta of ±4.59 points on the PTQ, ±2.51 on the GAD-7, and ±5.86 points on the ADS from T1-T3. Second, the use of anchor-based criteria was recommended for specifying practical meaningful change (e.g., Carrasco-Labra et al., 2021). Haase et al. (2021) used a slight improvement in the global impression of change assessed by the therapist as an anchor. On average, a meaningful improvement corresponded to a 30 % reduction in patient-reported symptoms of depression at the end of their therapy. Due to the lack of comparable studies for the other outcomes, we used the 30 % criterion for repetitive negative thinking, depression and anxiety to delineate practically-meaningful improvement and deterioration. Finally, for symptom-free status, published clinical thresholds for the CES-D for depression (Hautzinger et al., 2012) and GAD-7 for anxiety (Löwe et al., 2008) were used to indicate remission. As there is no validated cut-off for the PTQ, a proxy was used, defined as scoring more than two standard deviations (*SD*) below the study samples' mean of the PTQ-Scale at baseline, resulting in a PTQ score ≤ 23.29. For all response rates, the numbers needed to treat (NNT) for one additional beneficial outcome (NNTB) and for one additional harmful outcome (NNTH) in the intervention group, relative to the control group, were calculated (Higgins et al., 2024).

While results from ANCOVA indicate whether an intervention is significant on average, it remains an open question whether the intervention's effect may be significant for one specific subgroup, but not another. More specifically, it is of interest whether the intervention is significant for individuals with all levels of repetitive negative thinking or only for one or more subgroups; for example, only for those scoring above or below a critical value for repetitive negative thinking at baseline. The Johnson-Neyman procedure allows one to identify such a “region of significance” (Johnson and Fay, 1950), and was used to examine whether there were subgroups determined by their levels of repetitive negative thinking, depression, or anxiety at baseline for which the intervention's effect was significant for each respective variable.

## 3 Results

### 3.1 Participants

A total of 200 participants were randomized to either the intervention or control group. The flow of participants is shown in Fig. 1.

Baseline demographic characteristics and mental health status for all participants are summarized in Table 1. The average age of participants was 45.7 years (*SD* = 9.6). Roughly nine in ten reported being female (*n* = 177; 89.4 %), with almost half holding a university degree (*n* = 82; 41.4 %). Concerning mental health, 55 (27.8 %) reported subclinical symptoms of depression, while 88 (44.4 %) exceeded the threshold for clinical depression. The GAD-7 scores indicated moderate symptoms of anxiety in 65 participants (32.8 %), with severe anxiety symptom levels observed in a further 23 (11.6 %). More than half of the study sample reported some prior experience with psychotherapy (*n* = 110; 56 %), though only 28 (14.1 %) reported experience with any preventative mental health training.

**Table 1:** Characteristics of participants

	All [*N* = 198]				IG [*n* = 100]				WLC [*n* = 98]			
	*N*	%	*M*	*SD*	*N*	%	*M*	*SD*	*N*	%	*M*	*SD*
Age (years)			45.7	9.6			44.7	9.6			46.7	9.5
Sex												
Men	21	10.6			11	11.0			10	11.2		
Women	177	89.4			89	89.0			88	89.8		
Relationship												
Single	42	21.2			26	16.0			16	16.3		
Married or cohabiting	128	64.7			61	61.0			67	68.4		
Divorced or separated	24	12.1			11	11.0			13	13.3		
Widowed	4	2.0			2	2.0			2	2.0		
Children living in household	95	48.0			48	48.0			47	48.0		
Ethnicity												
White	168	84.7			86	86.0			82	83.7		
Black	1	0.5							1	1.0		
Hispanic	2	1.0			2	2.0						
Not reported	27	13.6			12	12.0			15	15.3		
University degree	82	41.4			46	46.0			36	36.7		
Employment status												
Full-time work	93	47.0			44	44.0			49	50.0		
Part-time work	64	32.3			31	31.0			33	33.7		
Family work	30	15.2			17	17.0			13	13.3		
Unemployed/seeking work	10	5.1			7	7.0			3	3.1		
On sick leave	1	0.5			1	1.0						
Symptoms of depression												
No elevated symptoms	55	27.8			23	23.0			32	32.7		
Subclinical symptoms	55	27.8			29	29.0			26	26.5		
Clinical symptoms	88	44.4			48	48.0			40	48.8		
Symptoms of anxiety												
Minimal level of anxiety	17	8.6			6	6.0			11	11.2		
Mild level of anxiety	93	47.0			47	47.0			46	46.9		
Moderate level of anxiety	65	32.8			37	37.0			28	28.6		
Severe level of anxiety	23	11.6			10	10.0			13	13.3		

Note. IG = intervention group; WLC = waiting-list control group. Symptoms of depression based on CES-D scores: No elevated symptoms = 0–16, subclinical symptoms = 17–22, clinical symptoms = 23–60. Levels of anxiety symptom based on GAD 7 scores: Minimal level = 0–4, mild level = 5–9, moderate level = 10–14, severe level = 15–21.

### 3.2 Missing data

Baseline data were available for all participants in the intervention group, but missing from 2 % (2/100) of the controls due to their withdrawal of written consent during the study. At six-weeks after randomization, 52.5 % of the participants (105/200) failed to provide data for repetitive negative thinking, but not for all other measures, due to a technical and organizational failure in the online assessment software. This technical problem was solved by the time of the three-month follow-up assessment, so data were missing from just 22.0 % of the participants (44/200) at three months follow-up. At six-months follow-up, when only participants in the intervention group were evaluated, 51.0 % (51/100) failed to provide questionnaire data for the primary outcome. Following the recommendation of Schafer and Graham (2002), Little's Missing Completely at Random-Test was employed. Results indicated that the assumption of data missing completely at random χ^2^ (503) = 553.39, *p* = .059 did not need to be rejected.

### 3.3 Primary outcome analysis

**Fig. 2:**
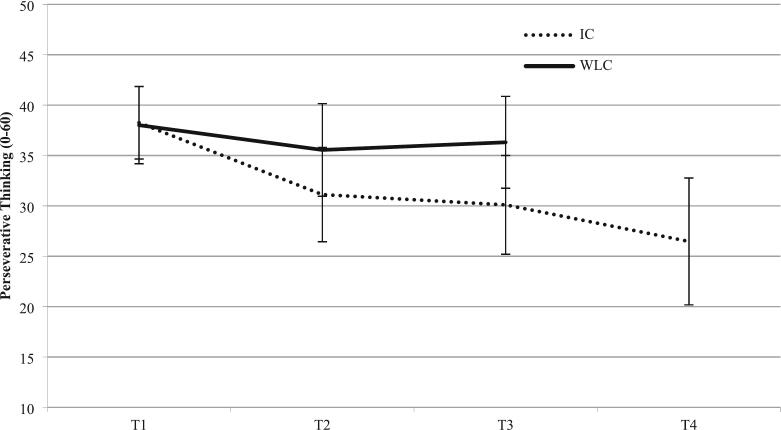
Development of repetitive negative thinking from baseline to six weeks to three-months and to six-months follow-up in the intervention group and waiting-list control group. Marginal means from the intention-to-treat sample. The scale ranges from 0 to 60. PTQ = Perseverative Thinking Questionnaire.

Table 2 lists the marginal means and standard deviations for repetitive negative thinking and all secondary outcome measures separately for all assessment time-points. Results of the respective AN(*C*)OVAs for all outcome measures are summarized in Table 3. Significantly lower PTQ scores were apparent in the intervention group versus controls three months after randomization, *F*(1, 155.04) = 18.02, *p* < .001. The corresponding difference was 6.21 points on the PTQ, representing an effect size of *d* = 0.66, 95 % [CI 0.37, 0.94]. For secondary comparisons, short-term effects at T2 also were significant (*F*(1, 169.84) = 9.17, *p* < .01) with a difference of 4.43 points on the PTQ, corresponding to *d* = 0.48, 95 % CI [0.19, 0.76]. Fig. 2 compares the two groups on the primary outcome measure over time.

**Table 2:** Marginal means and standard deviations of outcome variables at measurement points (intention-to-treat sample; N = 198).

	Baseline				6-weeks follow-up				3-months follow-up				6-months FU	
	IG		WLC		IG		WLC		IG		WLC		IG	
Outcomes variables	*M*	*SD*	*M*	*SD*	*M*	*SD*	*M*	*SD*	*M*	*SD*	*M*	*SD*	*M*	*SD*
Repetitive negative thinking	38.25	7.20	38.01	7.67	31.12	9.35	35.55	9.20	30.10	9.81	36.31	9.11	26.47	12.60
*Secondary outcomes*														
*Mental Health*														
Depression	22.67	8.12	21.09	8.91	16.28	7.79	20.26	9.58	17.73	8.94	21.58	9.59	16.96	13.62
Anxiety	9.56	3.79	9.14	4.21	6.99	3.55	8.74	4.14	7.00	4.15	8.51	3.79	6.96	6.18
Insomnia	11.58	5.44	11.61	5.32	9.69	5.84	10.92	5.88	9.28	5.50	10.49	5.41	9.20	6.82
Resilience ‡	29.97	5.68	29.69	6.11	31.93	5.99	30.47	6.08	32.27	6.55	30.20	5.80	33.97	8.15
*Personality*														
Gratitude ‡	19.05	3.19	19.29	3.48	20.72	3.05	19.85	3.21	20.33	3.32	19.83	3.67	20.82	3.67
Optimism ‡	11.45	2.16	11.51	2.05	11.44	2.15	11.39	2.49	11.3	2.36	11.50	2.45	11.68	2.38
*Social Resources*														
Social support ‡	26.49	5.24	26.06	5.02	27.04	4.83	25.97	5.60	27.42	4.66	26.28	5.31	26.64	4.96

Note. IG = intervention group; WLC = waiting-list control group; FU = Follow up.

‡ Higher scores indicate better outcomes.

**Table 3:** Results of the AN(*C*)OVAs and Cohen's *d*s (intention-to-treat sample) for between-groups effect post-test and at three months, and within-group effects at six months follow-up.

	Differences between study conditions				Differences within intervention group	
	6-week follow-up		3-months follow-up		6-month follow-up	
Outcome	*F*_*df*_	Cohen's *d* [95 % CI]	*F*_*df*_	Cohen's *d* [95 % CI]	*F*_*df*_	Cohen's *d* [95 % CI]
Repetitive negative thinking	9.17_1,169.84_⁎⁎	0.48 [0.20, 0.76]	18.02_1,155.04_⁎⁎⁎	0.66 [0.37, 0.94]	146.9_1,99_⁎⁎⁎	1.13 [0.81; 1.45]
*Secondary outcomes*						
*Mental Health*						
Depression	10.97_1,660.52_⁎⁎	0.46 [0.17, 0.74]	8.14_1,390.29_⁎⁎⁎	0.42 [0.13, 0.70]	28.71_1,99_⁎⁎⁎	0.51 [0.22; 0.79]
Anxiety	12.16_1,500.02_⁎⁎⁎	0.46 [0.17, 0.74]	8.51_1,463.77_⁎⁎	0.38 [0.10, 0.66]	33.4_1,99_⁎⁎⁎	0.49 [0.26; 0.72]
Insomnia	3.46_1,2156.5_+	0.21 [−0.07, 0.49]	3.41_1,389.51_+	0.22 [−0.06, 0.50]	20.19_1,99_⁎⁎⁎	0.38 [0.19; 0.57]
Resilience	6.41_1,1469.38_⁎⁎	0.24 [−0.04, 0.52]	8.58_1,231.34_⁎⁎	0.33 [0.05, 0.61]	66.4_1,99_⁎⁎⁎	0.53 [0.37; 0.69]
*Personality*						
Gratitude	5.90_1,1059.2_⁎	0.28 [0.00, 0.56]	1.4_1,414.71_^ns^	0.14 [−0.14, 0.42]	39.1_1,99_⁎⁎⁎	0.51 [0.33; 0.69]
Optimism	0.12_1,4754.29_^ns^	0.02 [−0.26, 0.30]	0.35_1,923.09_^ns^	0.08 [−0.36, 0.20]	0.98_1,99_^ns^	0.10 [−0.14; 0.35]

*Social Resources*						
Social support	4.96_1,708.81_⁎	0.20 [−0.07, 0,48]	3.56 _1195_⁎	0.23 [−0.05, 0.51]	0.12_1,99_^ns^	0.03 [−0.14; 0.20]

Note. CI = Confidence interval.

+ *p* ≤ .1;

⁎ *p* ≤ .05;

⁎⁎ *p* ≤ .01;

⁎⁎⁎ *p* ≤ .001.

### 3.4 Secondary outcome analysis

As shown in Table 3, ANCOVAs of the health-related outcomes depression, anxiety and resilience demonstrated significant between-group differences. Effect sizes for depressive symptoms ranged from *d* = 0.46 at T2 to *d* = 0.42 at T3, and for symptoms of generalized anxiety from *d* = 0.45 at T2 to *d* = 0.38 at T3. Effects in favour of the intervention group also were evident for resilience (*d* = 0.24 at T2, *d* = 0.33 at T3). Pertaining to personality- and resource-related variables, a significant difference between groups was found for gratitude at T2 (*d* = 0.28), but not for optimism, and for neither at T3. Likewise, the groups differed in their self-perceived level of social support at both T2 (*d* = 0.20) and T3 (*d* = 0.23).

### 3.5 Response and deterioration rates

From baseline to three-month follow-up, more participants in the intervention group improved, according to the criteria of reliable and clinically-meaningful improvement. More also achieved remission by scoring below outcome-specific thresholds. Corresponding numbers needed to treat ranged from NNTB = 2.74 for reliable improvement in repetitive negative thinking, up to NNTB = 8.46 for reaching symptom-free status for generalized anxiety. The NNTB for clinically-meaningful improvement, according to Haase et al. (2021), was 4.04 for depression. The number needed to treat for one additional beneficial outcome (NNTB) relates to a number of individuals and, therefore, should be interpreted as a whole number (Higgins et al., 2024). For example, the results suggest that three individuals are expected to take part in the gratitude training program to see one additional individual with reliable improvement in repetitive negative thinking, as compared to being on a waiting list with full access to care as usual.

Larger deterioration rates were found in the control group. However, deterioration was also experienced in the intervention group, ranging from 3 % for repetitive negative thinking up to 13 % reporting a ≥ 30 % increase in depressive symptoms. These results are summarized in Table 4.

**Table 4:** Response and deterioration rates for repetitive negative thinking, depression, and anxiety.

Outcome	Criterion	NNTB / NNTH	95 % CI	IG [n = 100]	WLC [n = 98]
Repetitive negative thinking	rel. Improv.	2.74	2.03–4.25	64	27
	rel. Det.	30.43	16.76 - ∞	11	14
	30 % improv.	4.39	2.94–8.63	34	11
	30 % det.	32.03	11.20 - ∞	3	6
	symptom-free^1^	4.80	3.20–9.60	29	8
Depression	rel. Improv.	3.75	2.56–7.09	46	19
	rel. Det.	10.58	5.00 - ∞	13	22
	30 % improv.	4.05	2.73–7.85	40	15
	30 % det.	11.86	5.29 - ∞	13	21
	symptom-free^2^	6.10	3.36–33.22	47	30
Anxiety	rel. Improv.	4.08	2.66–8.75	50	25
	rel. Det.	30.43	16.76 - ∞	11	14
	30 % improv.	2.98	2.17–4.74	53	19
	30 % det.	15.81	6.36 - ∞	10	16
	symptom-free^3^	8.46	4.00 - ∞	71	58

Note*.* CI = confidence interval; IG = intervention group; WLC = waiting-list control group; rel. Improv = reliable improvement; rel. Det = reliable deterioration; 30 % improv. = 30 % improvement; 30 % det. = 30 % deterioration. ^1^cut-offs for system-free status PTQ ≤ 23.29; ^2^CES-D ≤ 16; ^3^GAD-7 ≤ 9.

### 3.6 Long-term effects

Repeated-measures ANOVA in the intervention group confirmed a significant reduction in repetitive negative thinking from baseline (T1) to six-month follow-up (T4); *F*(1, 99) = 146.9, *p* < .001. Delta was 11.78 points on the PTQ, corresponding to a within-subject effect size of *d* = 1.13. Improvements in all health-related outcome measures were also maintained at six months (*p* < .001). Effect sizes ranged from *d* = 0.38 for insomnia to *d* = 0.51 for depression and resilience. Considering personality- and social-resource-related outcomes, a significant increase was found in participants' level of gratitude (*p* < .001). No significant changes were identified in the subjects' levels of optimism or perceived social support. Table 3 summarizes all long-term effects within the intervention group.

### 3.7 Subgroup identification

Moderation analysis using the Johnson-Neyman technique demonstrated a baseline PTQ score of 30.38 as the point of transition between a statistically-nonsignificant and statistically-significant effect on repetitive negative thinking three months after randomization. Similarly, significant effects of the intervention on depression were found for individuals scoring 18.85 points or more on the CES—D. Finally, participants scoring at least 7.54 points on the GAD-7 benefitted from the intervention in their symptoms of generalized anxiety disorder. Johnson-Neyman plots are provided in the supplementary material (S4).

### 3.8 Intervention and co-intervention uptake

Overall, 61 participants (61.0 %) in the intervention group completed all four modules of gratitude training, while 70 completed at least three. Participants claimed to have used the smartphone application an average of 4.92 days per week (*SD* = 2.4) at T3, and an average of 3.45 days weekly (*SD* = 2.3) at T4 (for a detailed description, see supplement S2).

The uptake of co-interventions provided by professionals or semi-professionals during the study phase was low and comparable between groups. In the intervention group, 3.0 % reported participating in other mental health training, 9.1 % in psychotherapy, and 14.6 % in counselling by semi-professionals (controls: 3.5 % training; 5.8 % psychotherapy; 13.0 % semi-professional counselling). However, in the intervention group, 39.0 % stated having used a self-help book for the same reason that had motivated them to participate in gratitude training. Noteworthy is that 71.7 % in the waiting-list control group reported using a self-help book, a difference of 32.7 % relative to those in the intervention group.

### 3.9 User satisfaction

Eighty of the 100 participants in the intervention group completed the CSQ-8 after the intervention, scoring a mean 25.64 points. While 95 % partly or fully agreed with the statement that the intervention was of high quality and 88.75 % indicated that they would recommend the intervention to a friend, only 26.25 % were fully satisfied with the extend of help they received from the training (60.00 % were partly satisfied). For a more detailed description of these results, see supplementary material (S5).

### 3.10 Sensitivity analyses

Additional analyses were conducted involving participants who completed the intervention (3 or 4 sessions) or the entire study, as shown in the study flow diagram (Fig. 1).

ANCOVAs demonstrated that participants who worked through 3 or 4 sessions of the gratitude program had significantly lower levels of repetitive negative thinking at T2, *F*(1, 89) = 28.97, *p* < .001 and T3, *F*(1, 145) = 44.55, *p* < .001, with stronger effects at T2, *d* = 0.86, 95 % *CI* [0.43, 1.35], and T3, *d* = 0.92, 95 % *CI* [0.58, 1.25] compared to ITT analysis.

Regarding study completers, significant effects were found in ANCOVAs indicating reduced repetitive negative thinking at T2, *F*(1, 81) = 25.68, *p* < .001 and T3, *F*(1, 156) = 43.69, *p* < .001, with higher effect sizes at T2, *d* = 0.89, 95 % *CI* [0.43, 1.35], and T3, *d* = 0.91, 95 % *CI* [0.58, 1.25], compared to the results of ITT analysis.

## 4 Discussion

The present study is the first to investigate the efficacy of a guided smartphone- and internet-based gratitude intervention for reducing repetitive negative thinking. For this purpose, a multicomponent gratitude training program was tested in a two-arm, pragmatic, randomized controlled trial. We found that this guided gratitude intervention was effective at reducing levels of repetitive negative thinking three months post randomization (*d* = 0.66), relative to being on a waiting list for the same program. Beneficial effects on repetitive negative thinking were also present after six weeks and were maintained for up to six months afterwards. These results were confirmed in sensitivity analyses, where effects appeared slightly larger. As repetitive negative thinking represents a transdiagnostic risk factor, it is notable that the gratitude training also had meaningful effects, in terms of reducing the symptoms of depression and generalized anxiety.

Putting these effects into context, a first observation was that the effects on repetitive negative thinking (*d* = 0.66) were stronger than expected in the sample size calculation (*d* = 0.40). It is also noteworthy that sensitivity analyses showed that those who completed three or all four sessions of the intervention benefited substantially more (*d* = 0.92), demonstrating the added potential of the intervention in a best-case scenario. Interestingly, the effects for this guided four-session version of the GET.ON gratitude were almost identical to the effects reported by Heckendorf et al. (2019) for the unguided five-session version. Unsystematic feedback from participants and eCoaches suggest that the individual sessions in the four-session version were too long and, therefore, less user-friendly. It remains speculative whether the advantages of guidance were offset by the disadvantages of a less user-friendly design of sessions. However, compared to the unguided gratitude mobile intervention (Kloos et al., 2022), the effects on repetitive negative thinking, depression and anxiety were consistently favourable, which might reflect an additional benefit of guidance. Besides the efficacy or costs for eCoaching (Nobis et al., 2018), providing human support serves as a safety measure, especially given that such an intervention seems to attract people with substantial baseline symptoms, including participants with a clinical level of depression. In cases of deterioration, participants have an established channel to seek help. Likewise, eCoaches could actively monitor negative developments and contact participants (cf., Sander et al., 2020). Although deterioration rates in the present study fall within the range observed for other internet interventions (e.g., Harrer et al., 2024), labelling interventions such as gratitude as “positive” could be misleading and might explain why negative effects in positive interventions are rarely addressed (cf., Bohlmeijer and Westerhof, 2021; Hendriks et al., 2020).

Effects of the present gratitude intervention compared favourably with results reported for the meta-analysis published by Spinhoven et al. (2019), demonstrating that treatments for depression reduce repetitive negative thinking effectively by *g* = 0.48, on average, in clinical samples. Interestingly, the current effects were only marginally weaker when compared to rumination-focused cognitive behavioural therapy (CBT, *g* = 0.76), were in line with effects reported for cognitive control training (*g* = 0.62) and traditional CBT (*g* = 0.57), and were slightly stronger than for mindfulness-based cognitive therapy (*g* = 0.42). Taken together, these results suggest that digital multicomponent gratitude interventions may expand the options of interventions for reducing repetitive negative thinking.

Besides repetitive negative thinking, the effects of the gratitude intervention we observed on depression (*d* = 0.42) exceeded the threshold for clinically-meaningful effects proposed by Cuijpers et al. (2014) of a standardized mean difference (SMD) of *SMD* = 0.24. Compared with transdiagnostic treatments in clinical samples, the effects for the gratitude intervention were smaller for depression (*d* = 0.42 vs. *g* = 0.61) and for anxiety (*d* = 0.38 vs. *g* = 0.54) (Cuijpers et al., 2023). However, such a comparison is potentially problematic, due to differences in the clinical status of the studies` participants. While the present study was conducted from a prevention perspective and, as such, also included individuals with no or only subclinical symptoms, the above-mentioned meta-analyses (Cuijpers et al., 2023; Spinhoven et al., 2019) solely included clinical samples, in which larger effects are more likely (e.g., Carr et al., 2020). Looking at meta-analyses evaluating internet interventions targeting the general population (Deady et al., 2017), the present effect sizes compare favourably for both depression (*d* = 0.25) and anxiety (*d* = 0.31) and are consistent with the meta-analysis by Reins et al. (2021) investigating internet interventions designed to target subclinical depression (*g* = 0.39). Hence, multicomponent gratitude interventions like GET.ON gratitude could serve as a transdiagnostic intervention for individuals approached in the general population with elevated symptoms. On the other hand, evidence for individuals in treatment or other clinical contexts remains missing.

The meta-analysis by Cregg and Cheavens (2021) on gratitude interventions generated effect estimates for depression and anxiety of *g* = 0.17 and *g* = 0.16, respectively. The authors concluded that gratitude interventions should not be recommended to individuals seeking help for depression or anxiety, preferring internet interventions for depression. However, the meaningful effects on depression (*d* = 0.42) and anxiety (*d* = 0.38) observed in the present study, as well as results from Heckendorf et al. (2019), suggest that Cregg and Cheavens' conclusions might be premature and should be regarded as merely one snapshot in the evolution of effective gratitude interventions. Instead, it seems more important to focus on the design, content, process of optimization, and target groups of gratitude interventions. For example, single gratitude exercises, like a diary, may be useful and effective from a universal prevention perspective, where even small effects are practically meaningful for public health promotion (Matthay et al., 2021). The present gratitude intervention, however, is a multicomponent program incorporating a multitude of exercises addressing the cognitive, emotional, and behavioural facets of gratitude. Hendriks et al. (2020) appear to concur by differentiating between single and multicomponent positive psychological interventions.

Apart from the encouraging effects in the present study that were comparable to interventions for depression, the recommendation by Cregg and Cheavens (2021) to use internet interventions for depression instead of for gratitude could be double-edged. In light of the low uptake rates of anti-depression interventions, Cuijpers (2021) introduced the paradigm of the indirect prevention and treatment of depression. With “direct” interventions labelled “anti-depressive”, it is argued that the label itself could be perceived as stigmatizing and thereby limit the interventions' uptake and reach. Accordingly, Cuijpers (2021) submitted the need for complementary “indirect” interventions that focus on features perceived as positive, to improve mental health in the population. Results from different studies support the notion that gratitude interventions are more widely accepted in the general population (Folk and Dunn, 2023) and are preferred when participants can choose between different evidence-based exercises (Heckendorf et al., 2022). Therefore, when designing comprehensive strategies to reduce the burden of depression in the general population, established, direct anti-depressive interventions could be complemented by multicomponent gratitude interventions or other evidence-based indirect interventions like stress-management programs (Harrer et al., 2024) or further positive psychological interventions (Hendriks et al., 2020).

This also may be described in terms of the diversity of interventions. It may be necessary to increase the diversity of interventions to seriously account for the increasing diversity in societies and, thereby, offer a promising route to increase the overall reach of effective interventions in the population. Nevertheless, more evidence for multicomponent gratitude interventions is needed to support this strategy. Likewise, evidence is needed that combining direct and indirect interventions increases the overall reach of interventions and, by doing so, reduces the burden of depression in the population, as proposed by Cuijpers (2021).

The present study also provides a better description of the population that is likely to benefit from such multicomponent gratitude interventions. Results of Johnson Neyman analysis demonstrated that individuals with moderate subthreshold or more severe symptoms benefitted with significantly-reduced repetitive negative thinking, depression, and anxiety. Conversely, it appears that the present multicomponent gratitude intervention seems not to be beneficial for all, as there is no indication that individuals with minor or no complaints will experience mental health benefits. Similar results were reported by Behrendt et al. (2020), suggesting that the traditional distinction between non-clinical and clinical samples might not capture the characteristics of individuals for whom multicomponent internet interventions work well and can be recommended. The present results empirically confirm earlier assumptions by Cregg and Cheavens (2021), which suggest that the severity of psychopathology moderates the effect of gratitude interventions.

Effects on further secondary outcomes were mixed. Whereas the intervention exhibited beneficial effects for resilience, the results confirm other findings that gratitude interventions have little or no effects on the personality trait of optimism (Heckendorf et al., 2019). While Heckendorf et al. (2019) found consistent effects on dispositional gratitude for the GET.ON gratitude intervention, the present results support these findings only partially. Effects on trait gratitude are especially of interest, as recent research has found dispositional gratitude to be associated with increased longevity (Chen et al., 2024). However, Jackson et al. (2012) have emphasized the importance of prolonged changes in trait-relevant behaviour, and the present four-session intervention might have been too short to yield more pronounced effects on personality. Finally, significant effects on perceived social support agree with the assumptions of Algoe (2012), outlining the function of gratitude in strengthening social relationships with responsive others.

### 4.1 Limitations and further directions

Despite the overall encouraging findings of the current study, several limitations should be considered carefully. First, as almost nine out of ten participants were female, generalization beyond females must be considered with caution. Similarly, Cregg and Cheavens (2021) reported that 16 out of 27 gratitude interventions studies included from 80 % to 100 % female participants; however, they observed no moderating effect of sex. Nevertheless, when designing an overall public mental health promotion strategy employing “indirect” interventions, further approaches are needed to address mental health in males. Second, there was an indication of treatment diffusion bias, as over 70 % of the participants in the control group actively sought help from self-help literature while waiting. The observed self-initiated activation of the control group contrasts with earlier speculations that participants on waiting lists reduce their self-help activities (Furukawa et al., 2014). Single gratitude exercises, especially the diary, are salient in the media (Folk and Dunn, 2023), found in self-help books, and easy to apply. While the increased self-help activities limit our study's internal, it strengthens its external validity. Consequently, differences in outcomes between study arms may decrease, leading to the potential underestimation of effects.

Third, to mimic an indicated prevention approach, participants had to experience elevated levels of repetitive negative thinking. Further studies are needed that mimic the program implementation provided in this study to determine how the program might work for more universal prevention.

Fourth, although effects were found for the transdiagnostic risk-factor repetitive negative thinking and the assumed pathologies of depression and anxiety, the study was not designed to test the respective mechanisms behind these benefits. This said, Heckendorf et al. (2019) have reported evidence for a proposed mechanism (cf. Domhardt et al., 2021).

Fifth, the results for this multicomponent intervention compare favourably with previous meta-analyses on primarily single-component interventions. However, such comparisons based on data from different studies are indirect, and direct head-to-head comparisons are needed to draw firm conclusions about the different levels of efficacy. Moreover, adding complexity to interventions makes it harder to identify the active components.

Sixth, the criteria for defining response rates remain debatable. For example, the results of Bauer-Staeb et al. (2021) suggest that the 30 % criterion may be too high for those with only mild symptoms. Furthermore, the assumption of symmetry using the same criterion for specifying improvement and deterioration may be inadequate. According to the “bad is stronger than good” principle (Baumeister et al., 2001), smaller changes might indicate meaningful worsening.

Finally, some process evaluation with in-depth qualitative interviews of participants would have been helpful to optimize the intervention (e.g., Behrendt et al., 2023). Although the participants' overall satisfaction was comparable to that expressed for similar interventions (Boß et al., 2016), our results indicate room for improvement and two improvements were implemented: The mobile component of the intervention was not free of initial bugs that might have limited the program's overall efficacy. Also, after the trial, the individual sessions were shortened, and the content distributed across five instead of four sessions. Considering the optimization of gratitude interventions in general, one potential reason for the pessimistic evaluation of gratitude interventions (e.g., Cregg & Cheavens, 2021) could be poor intervention descriptions in previous gratitude research, making it difficult for intervention designers to learn from prior experiences and systematically improve the design and delivery of gratitude interventions.

## 5 Conclusions

Despite reserved conclusions on the effectiveness of gratitude interventions (Davis et al., 2016; Cregg & Cheavens, 2021), the present study suggests that a next generation of multicomponent gratitude interventions might be effective at reducing the transdiagnostic risk-factor of repetitive negative thinking, as well as the symptoms of depression and anxiety to a clinically-meaningful extent. Nevertheless, results also show that negative health changes and developments while participating in positive interventions exist and should be taken into account; for example, by providing personal support. Future studies should consider that people on waiting lists might increase their help-seeking activities, a phenomenon that is particularly important when (parts of) the interventions of interest are already known to the public, easily accessible, and applicable in self-help. Though further research remains necessary, multicomponent gratitude interventions broaden the options of transdiagnostic interventions, fit well with the idea of the new paradigm of indirect prevention and treatment of depression (Cuijpers, 2021), and seem particularly beneficial for individuals experiencing moderate-subthreshold or more pronounced symptoms.

## CRediT authorship contribution statement

DL, HF and BS developed the intervention. DL, HF, MB, HR and DDL were involved in study design. DL was responsible for data collection. DL and LK were responsible for analysis, and interpretation. DL wrote the first draft of the manuscript. All other authors edited the text and contributed to the writing. All authors have approved the final version of the manuscript.

## Funding

The 10.13039/501100000780European Union funded this study, under project number: EFRE: CCI 2007DE161PR001. This publication was funded by the German Research Foundation (DFG).

## Data and intervention availability

Datasets are available on request from the first author. The raw data supporting the conclusions of this manuscript will be made available by the authors, without undue reservation, to any qualified researcher. Access to the intervention is available on request from the first author.

## Declaration of competing interest

The authors declare the following financial interests/personal relationships which may be considered as potential competing interests: Dirk Lehr, Matthias Berking, David Daniel Ebert reports financial support was provided by European Union. Matthias Berking has served as a consultant on the scientific advisory boards of GET.ON institute and mentalis GmbH, used to be shareholder of GET.ON institute, and is currently shareholder of mentalis GmbH. David Daniel Ebert has served as a consultant to/on the scientific advisory boards of Sanofi, Novartis, Minddistrict, Lantern, Schoen Kliniken, Ideamed and German health insurance companies (BARMER, Techniker Krankenkasse) and a number of federal chambers for psychotherapy. He is also shareholder of "GET.ON Institut für Online Gesundheitstrainings GmbH für Gesundheitstrainings online GmbH" (HelloBetter), which aims to implement scientific findings related to digital health interventions into routine care. All other authors, declare that they have no known competing financial interests or personal relationships that could have appeared to influence the work reported in this paper.

## Appendix A Supplementary data

Supplementary material Image 1
