# Supplementary material for: Effectiveness of a guided multicomponent internet and mobile gratitude training program - A pragmatic randomized controlled trial
Source: Internet Interv. 2024 Nov 12;38:100787. doi: 10.1016/j.invent.2024.100787 (PMC11615527; doi:10.1016/j.invent.2024.100787)
Supplement: Supplementary file 1 — Supplementary material [file mmc1.docx]

| Effectiveness of a Guided Multicomponent Internet and Mobile Gratitude Training - a Pragmatic Randomized Controlled Trial  Supplementary Material |
| --- |

Content

- S1. Upward spiral of gratitude and wellbeing
- S2. Description of GET.ON gratitude
- S3. Reliability of measures
- S4. Johnson-Neyman plots for repetitive negative thinking, depression and anxiety
- S5. User satisfaction

**S1.** Upward spiral of gratitude and wellbeing. Working model used in the GET.ON interventions with the aim to place the various exercises in an overall framework that is easy to understand for participants.


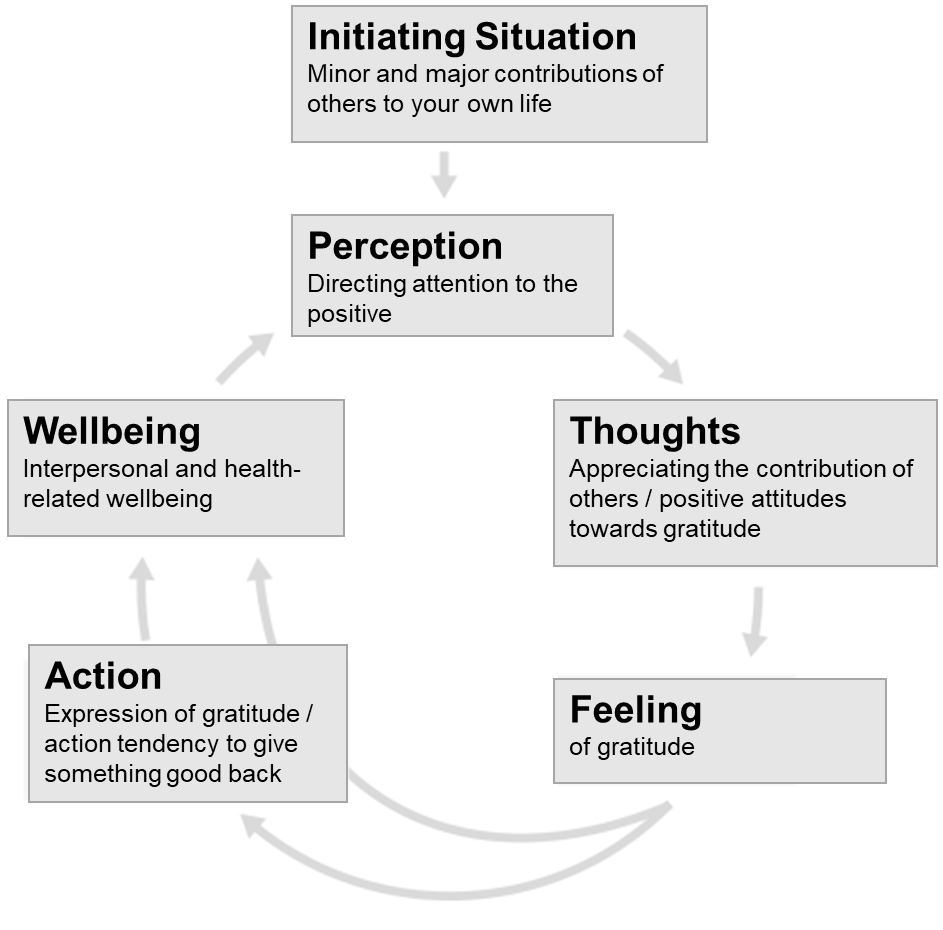


**S2.** Description of GET.ON gratitude

The intervention was designed according to the upward spiral of gratitude and well-being framework (S1). Firstly, individuals need to pay attention and notice a potentially positive event or experience (Awareness). Secondly, the event or experience has to be appreciated, via different cognitive appraisals like interpreting it as helpful, well meant or valuable (cognition). Appreciation activates the emotional experience of gratitude (emotion). The emotion of gratitude may either evoke the desire to express gratitude towards the benefactor or others (action), thereby increasing well-being, or it can foster positive affectivity (well-being) directly. The model was used as a framework to integrate 17 different gratitude exercises into a comprehensive multicomponent gratitude intervention. Exercises like gratitude journaling, writing a letter expressing gratitude, and gratitude visits were adopted from the literature. Additionally, established therapeutic exercises were modified to enhance gratitude.

The first and second modules both focus on exercises to increase awareness of positive events and experiences. At the beginning, participants can conduct short experiments on selective attention demonstrating the selectivity of perception, emphasizing the importance of consciously directing attention towards the positive. In another exercise, participants are encouraged to think about all life domains and specify for what they are grateful. In a ‘milestones of gratitude’ exercise, they are asked to reflect on milestones of gratitude throughout their lifetime and visualize those milestones. The smartphone app is introduced for raising awareness of the positive in daily life (see below).

Intensifying the emotional experience of gratitude is a major goal of the second module. To assist with this, an imaginary journey is developed, and further exercises are introduced to help users learn how to utilize sensory modalities and body sensations to intensify the emotion of gratitude. Specifically, participants can learn to support the emotional experience of gratitude by using (inner) pictures, playing or listening to music related to a certain moment of gratitude, using pleasant smells associated with moments of gratitude, or paying attention to bodily sensations associated with gratitude. These exercises can be repeated over subsequent weeks. In an educational section, participants learn to challenge dysfunctional beliefs about the relationship between gratitude, rumination and worry.

In the third module, participants are asked to identify dysfunctional cognitions that hamper their experience of gratitude; for example: “I can only be grateful if the other person has come up with the idea of doing something good for me all by themselves.” Subsequently, different exercises to achieve cognitive restructuring are offered, to help individuals develop and practice a gratitude-friendly attitude; for example, by conducting a behavioural experiment. As part of both this and the fourth module, participants are encouraged to express their gratitude in creative ways. Methods gleaned from the literature, such as writing a gratitude letter or making a gratitude visit, are especially highlighted. However, participants also may either choose from a list of more than 100 actions to express their gratitude or identify their own way to do so. In another exercise, participants can set a period of time during which their goal is to “catch” someone doing something good for them.

The fourth module also evaluates the progress that participants have achieved and asks them to identify those exercises that were especially helpful. Finally, they are asked to make a plan for the future and specify which exercises they will practice in daily life after they completed the training.

It should also be noted that the intervention includes a special section on potential negative effects of gratitude, pointing out limitations and dysfunctional applications of gratitude exercises. This includes, for example, using gratitude to avoid feeling justified sadness, disappointment or anger, or to avoid active problem-solving. In another exercise, the aim is to reflect on moments of gratitude over the last week and to examine whether these moments also were associated with negative feelings like shame or indebtedness.

The web-based modules are complemented by daily exercises on the smartphone gratitude app, which has two key functions. First, participants are asked to notice positive aspects in their everyday life by writing a brief note or (preferably) taking a picture related to their positive experience. Second, the app provides the opportunity for users to participate in a brief period of gratitude-focused meditation each evening. All gratitude notes and pictures they have taken throughout the day are presented on one screen. The participants are then asked to reflect on those experiences, focusing on the perceived emotions of gratitude. Additionally, they activate reminders (gratitude nudges), that are sent according to their own preferences. The reminder asks participants to check if there is something right now that is worth being recorded, by either writing a note or taking a picture.

**S3.** Reliability of measures

| *Internal consistency in main publication and the present study and test-retest reliability of measures* | | | | | | | | | | | | |
| --- | --- | --- | --- | --- | --- | --- | --- | --- | --- | --- | --- | --- |
|  | M | T1 | T2 | | | T3 | T4 | R_t1-t2_ | | | R_t1-t3_ | R_t1-t4_ |
| Repetitive negative thinking^1^ | .95 | .89 | .94 | | | .95 | .95 | .47 | | | .49 | .19 |
| *Secondary outcomes* | | | | | | | | | | | | |
| *Mental Health* | | | |  |  | | | |  |  | | |
| Depression^2^ | .89-.92 | .87 | .90 | | | .91 | .94 | .47 | | | .44 | .08 |
| Anxiety^3^ | .89 | .87 | .89 | | | .90 | .94 | .56 | | | .59 | .39 |
| Insomnia^4^ | .74 | .82 | .88 | | | .86 | .87 | .67 | | | .70 | .56 |
| Resilience^5^ | .85 | .81 | .85 | | | .87 | .90 | .78 | | | .74 | .71 |
| *Personality* |  | | |  |  | | | |  |  | | |
| Gratitude^6^ | .82 | .69 | .65 | | | .74 | .60 | .66 | | | .70 | .63 |
| Optimism^7^ | .76 | .72 | .78 | | | .74 | .80 | .37 | | | .32 | .24 |
| *Social Resources* | | | | | | | | | | | | |
| Social support^8^ | .63-.83 | .91 | .91 | | | .91 | .90 | .81 | | | .80 | .62 |

M = internal consistency provided by the main publication for the measure as described in the methods section. ^1^: Ehring et al., 2011; ^2^: Hautzinger et al., 2012; ^3^: Löwe et al., 2008; ^4^: Bastien et al., 2001; ^5^: Connor & Davidson, 2003; ^6^: McCullough et al., 2002; ^7^: Scheier et al., 1994; ^8^: Schulz & Schwarzer, 2003

**S4.** Johnson-Neyman plots for repetitive negative thinking, depression and anxiety

| 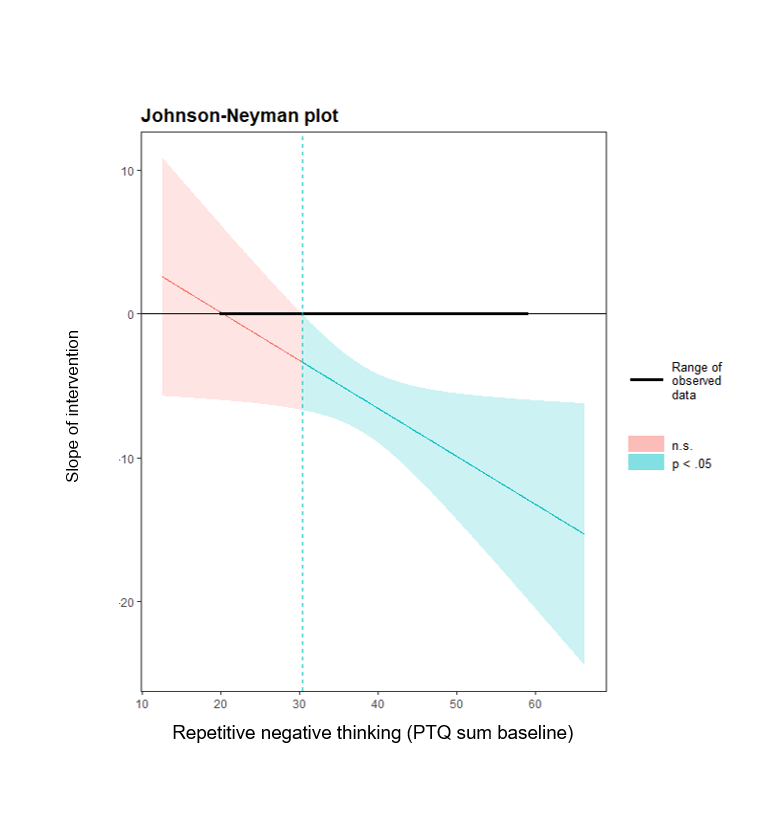 *Note. PTQ = Perseverative thinking questionnaire; Slope of intervention is p < .05 when PTQ sum baseline ≥ 30.38*  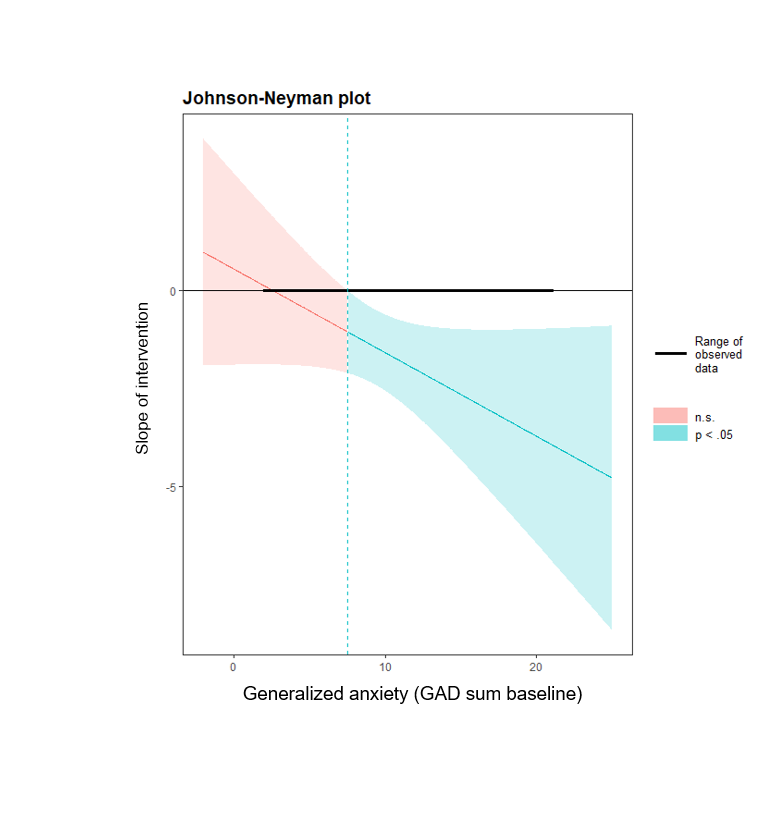 *Note. GAD = German Version of the Generalized Anxiety Disorder Questionnaire; Slope of intervention is p < .05 when GAD sum baseline ≥ 7.54* | 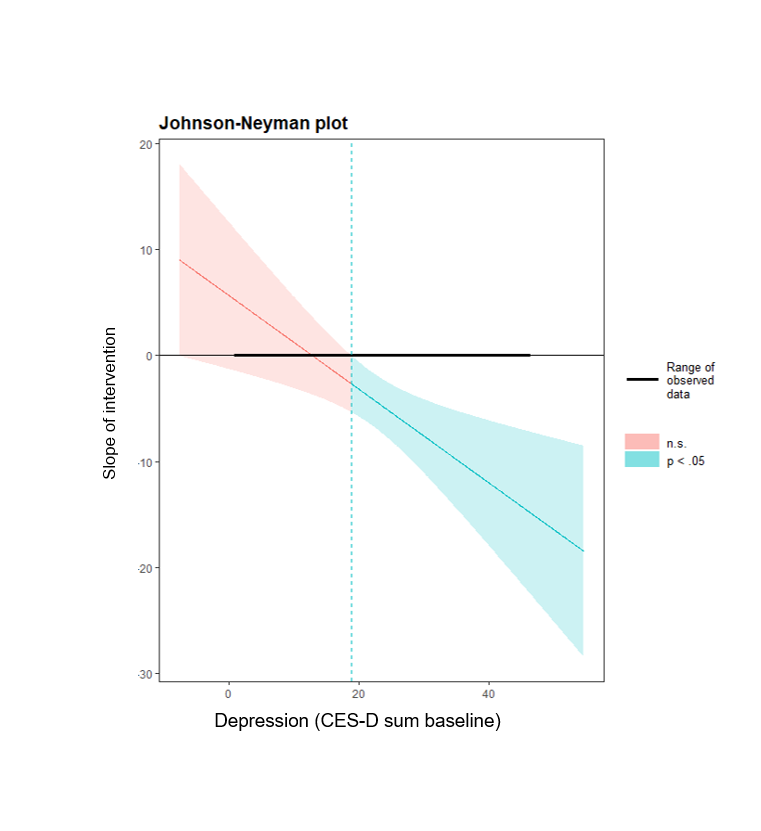 *Note. CES-D = German Version of the Centre for Epidemiological Studies Depression Scale; Slope of intervention is p < .05 when CES-D sum baseline ≥ 18.85* |
| --- | --- |

**S5**. User satisfaction.

| CSQ-8 Item^a^ | *M* | *SD* | Agreement^b^  *n %* | |
| --- | --- | --- | --- | --- |
| Quality of the training | 3.30 | 0.64 | 76 | 95.00 |
| Received the training I wanted | 3.16 | 0.70 | 72 | 90.00 |
| Training met my needs | 3.04 | 0.74 | 66 | 82.50 |
| Would recommend the training to a friend | 3.41 | 0.79 | 71 | 88.75 |
| Received the amount of help I wanted | 3.09 | 0.72 | 69 | 86.25 |
| Training helped me to deal more effectively with my problems | 3.06 | 0.83 | 63 | 78.75 |
| Generally satisfied with the training | 3.33 | 0.78 | 71 | 88.75 |
| Would use the training again | 3.25 | 0.88 | 65 | 81.25 |

*Notes. ^a^ Not at all (1), Rather not (2), Yes in parts (3), Yes totally true (4); ^b^ responded with ≥3; n=80*
